# Antibacterial and Antioxidant Activity of Essential Oil Terpenes against Pathogenic and Spoilage-Forming Bacteria and Cell Structure-Activity Relationships Evaluated by SEM Microscopy

**DOI:** 10.3390/molecules191117773

**Published:** 2014-11-03

**Authors:** Hatice Zengin, Ayse H. Baysal

**Affiliations:** Department of Food Engineering, Izmir Institute of Technology, Urla, Izmir 35437, Turkey; E-Mail: haticeyzengin@gmail.com

**Keywords:** essential oils, terpenes, GC/MS, antibacterial activity, synergism, action mechanisms, SEM

## Abstract

The antibacterial activity and antioxidant effect of the compounds α-terpineol, linalool, eucalyptol and α-pinene obtained from essential oils (EOs), against pathogenic and spoilage forming bacteria were determined. The antibacterial activities of these compounds were observed *in vitro* on four Gram-negative and three Gram-positive strains. *S. putrefaciens* was the most resistant bacteria to all tested components, with MIC values of 2% or higher, whereas *E. coli* O157:H7 was the most sensitive strain among the tested bacteria. Eucalyptol extended the lag phase of *S*. Typhimurium, *E. coli* O157:H7 and *S*. *aureus* at the concentrations of 0.7%, 0.6% and 1%, respectively. *In vitro* cell growth experiments showed the tested compounds had toxic effects on all bacterial species with different level of potency. Synergistic and additive effects were observed at least one dose pair of combination against *S*. Typhimurium, *E. coli* O157:H7 and *S. aureus*, howeverantagonistic effects were not found in these combinations. The results of this first study are encouraging for further investigations on mechanisms of antimicrobial activity of these EO components.

## 1. Introduction

Natural products, such as essential oils (EOs) which are produced by the secondary metabolism of herbs and/or spices and their constituents have uses in human consumption as functional food (nutraceuticals, biopolymers), food additives (flavourings, antioxidant and antimicrobial), medicines (pharmaceuticals, therapeutic products), nutritional supplements (dietary supplements, culinary) and the manufacture of cosmetics (perfume/fragrances, aromatherapy, hair and skin care). Due to their antiradical, antioxidant, anti-inflammatory and antimicrobial properties antioxidants are effective for inhibiting different human diseases. Thus, evaluation of bioactive compounds, prominently polyphenols, from natural plant sources, including vegetables, fruits, herbs and spices has shown an increasing trend. Plant EOs have antibacterial, antioxidant and antimutagenic activities, and potential beneficial health effects. These generally recognized as safe (GRAS) natural substances inhibit lipid oxidation in foods, and thus offer the promise of providing natural food additives to food products.

The antimicrobial activity of some EO components was tested on various microorganisms and they were found to be effective against almost all bacterial species [1,2,3,4], however the mechanism of the antimicrobial activity in the case of EOs is still not entirely clear. Although EOs in plants are generally the mixtures of abundant components [5], there are not many studies related to the activities of the EO components and their mechanisms of action. Moreover, in the literature few studies have reported the synergistic effects achieved using EO combinations at sufficiently low concentrations and as a result reducing the negative sensory impact [6,7].

The antimicrobial activity of several EOs has been attributed to the presence of specific phenolic compounds [8,9,10,11,12,13], therefore in this study, the antimicrobial activity of four terpene molecules found in some EOs has been evaluated on different species of food pathogens and spoilage bacteria. Moreover, as EOs and their components are known to disrupt the cellular membranes [14,15], their toxicity has been evaluated using *in vitro* bacterial cell model.

The purpose of this study was thus to determine the antibacterial activities of some terpene EO components (α-terpineol, linalool, eucalyptol and α-pinene) individually as well as in combination to determine their interactions at lower doses against common pathogenic and spoilage food-related bacteria by *in vitro* approaches. Also alterations in microbial cell membranes and structures were evaluated by using SEM microscopy in order to understand their antibacterial mode of action.

## 2. Results and Discussion

### 2.1. Determination of Antioxidant Acitivity by FRAP and DPPH Method

Total antioxidant activities of the EO constituents determined with both the FRAP and DPPH methods are given in Table 1. The overall antioxidant activity of α*-*terpineol was the strongest, followed in descending order by linalool and eucalyptol (Table 1). Eucalyptol showed weak antioxidant activity by the FRAP method, and almost no free radical scavenging activity with the DPPH method. The relationship and antioxidant activity are also highly influenced by the different assay methods used [16]. Both results obtained from FRAP and DPPH assays have nearly the same outcome. Some authors also reported that there were differences in the results obtained from these two assays [17,18], making it difficult to compare the results of different methods used to determine antioxidant activity in the literature, so any interpretation of antioxidant activity may require a combination of different methods..

**Table 1:** Antioxidant Activity of EO components (terpenes) measured by the FRAP and DPPH methods.

EO Component	FRAP (mmol Trolox mL^−1^)	DPPH (IC_50_ µL·mL^−1^)
Eucalyptol	0.58 ± 0.22	NA
α-terpineol	1.23 ± 0.56	433.97 ± 13.69
Linalool	1.20 ± 0.27	325.05 ± 20.19

NA: Not Active.

In a study where the antioxidant activity of the components of *Salvia tomentosa* Miller (Lamiaceae) EOs was examined, terpinene-4-ol, 1,8-cineole, camphor, borneol, *p*-cymene, α-pinene and β-pinene showed no activity [19]. In order to determine the antioxidant nature of *Achillea millefolium* subsp. *millefolium* Afan. (Asteraceae) oil, its main components, which account for 60.7% of the total, e.g., eucalyptol, camphor, β-pinene, borneol, terpinen-4-ol and α-pinene, were all tested individually and none exhibited antioxidative activity in any of the assays employed [20]. The reason that EOs showed much more activity than their constituents alone can be attributed to the high percentages of the main components, synergy among the different oil constituents or to microcomponents acting as pro-oxidants [21]. However, antioxidant activity of eucalyptol and terpinen-4-ol was previously reported using the aldehyde/carboxylic acid assay and lipid peroxidation methods [22]; with prolonged incubation periods (30 days and 18 h, respectively) in contrast to experimental procedure employing 30–60 min incubation period [20].

Our findings for *α*-terpineol (a monoterpene alcohol) and linalool (an acyclic terpene alcohol) showed that there were very weak, and eucalyptol had almost no antioxidant activity. Oxygenated monoterpenes, especially thymol and carvacrol, have high antioxidant activity. Although, monoterpene hydrocarbons may be considered as active antioxidants, none are stronger than oxygenated monoterpenes. Sesquiterpene hydrocarbons and their oxygenated derivatives have very low antioxidant activity [23].

### 2.2. Determination of Antibacterial Activity and Minimal Inhibitory Concentration (MIC)

**Figure 1:**
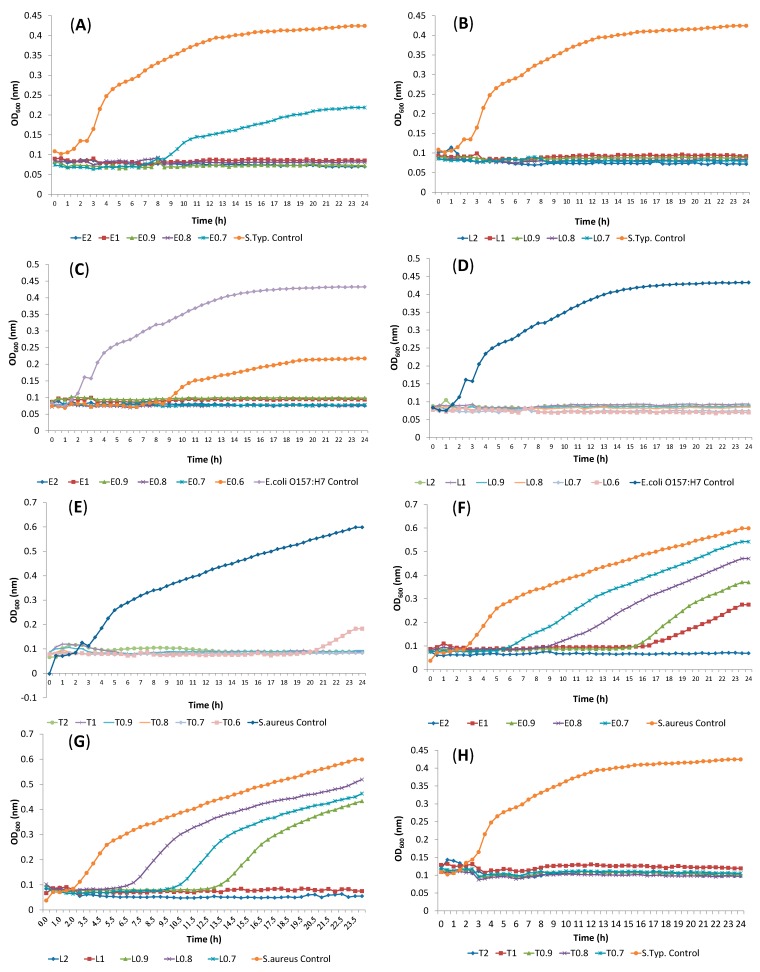
Inhibition of cells treated with (**A**) eucalyptol, *S*. Typhimurium; (**B**) linalool, *S*. Typhimurium; (**C**) eucalyptol, *E. coli* O157:H7; (**D**) linalool, *E. coli* O157:H7; (**E**) α-terpineol, *S. aureus*; (**F**) eucalyptol, *S. aureus*; (**G**) linalool, *S. aureus*; (**H**) α-terpineol, *S*. Typhimurium. (T: α-Terpineol, L: Linalool, E: Eucalyptol).

Before the interaction study, each EO constituent (terpene) was subjected to a broth microdilution assay to determine the MIC values for *S. aureus*, *S.* Typhimurium, *E. coli* O157:H7, *S. liquefaciens*, *C. divergens*, *L. innocua* and *S. putrefaciens*. Figure 1 shows the concentration ranges during the study and MIC values of the examined components for *S.* Typhimurium, *E. coli* O157:H7 and *S. aureus*.

Twenty four h observation of bacterial growth indicated that the MIC values of α-terpineol were 0.6% for *E. coli* O157:H7, *S. liquefaciens*, *C. divergens* and *L. innocua* (Table 2). *S. aureus* and *S.* Typhimurium were inhibited with the concentration of 0.7% of α-terpineol. *S. aureus*, *L. innocua* and *S. liquefaciens* showed the same MIC value of 1% for linalool, while *E.coli* O157:H7 had a MIC value of 0.6% following by *S.* Typhimurium (0.7%). A significant retardation was observed in *S. aureus* growth when 0.6% α-terpineol was used (Figure 1F).

It is obvious that all tested bacteria were comparably resistant to eucalyptol, with MIC values of 2 or >2. The most sensitive bacteria to eucalyptol were *E. coli* O157:H7 (0.7%) and *S.* Typhimurium (0.8%). Eucalyptol elongated the lag phase of *S*. Typhimurium, *E. coli* O157:H7, *S*. *aureus* at the concentration of 0.7%, 0.6% and 1% respectively (Figure 1A,C,F). *S. putrefaciens* was the most resistant bacteria to all tested components, with MIC values of 2% or higher, whereas, *E. coli* O157:H7 was the most sensitive strain among the tested bacteria. All tested bacteria were resistant to α-pinene with the MIC values above the concentration of 2% (data not shown).

**Table 2:** Antimicrobial Activity of EO Components Expressed as MIC (%) Determined by Broth Microdilution.

Bacteria	MIC (%) for EO Components (*v*/*v*) *		
	α-Terpineol (T)	Linalool (L) %	Eucalyptol (E) %
**Gram Positive Bacteria**			
*Staphylococcus aureus*	0.7	1	2
*Carnobacterium divergens*	0.6	2	2
*Listeria innocua*	0.6	1	>2
**Gram Negative Bacteria**			
*Shewanella putrefaciens*	2	>2	>2
*Serratia liquefaciens*	0.6	1	>2
*E.coli* O157:H7	0.6	0.6	0.7
*Salmonella* Typhimurium	0.7	0.7	0.8

*: Concentration Ranges: 0.03–2.

Linalool, eucalyptol (1,8-cineole), α-terpinol and α-pinene were previously tested for antimicrobial activity and MIC and MBC (minimum bactericidal concentration) values were also determined by other authors [3,4,9,24,25,26,27,28,29,30,31,32]. The range of concentrations of linalool reported to inhibit the growth of microorganisms (MIC) were >1000 μg·mL^−1^ [9] in solid (visible growth) and in liquid media at 2.5 mg·mL^−1^ [28], >13.3–3.33 μL·mL^−1^ [29] and >20–10 μL·mL^−1^ [30]. 1,8-Cineole (eucalyptol) is the major oxide found in EOs. It is the main component of eucalyptus oil and may also be present in high quantities in rosemary oil. MICs for 1,8-cineole include 0.25% to 0.5% (*v*/*v*), 207.5 mM (3.2%), 2.8 to 5.6 mg·mL^−1^ (0.28% to 0.56%) and 1.25 to 2 mg·mL^−1^ (0.12% to 0.2%) for a range of bacteria such as *E. coli*, *S. aureus* and *Bacillus* species. However, one study reported no inhibition for *B. cereus* and *E. coli* at 207.5 mM (3.2%) [4]. Linalool showed bacteriostatic properties with MIC ≤ 0.2 μL·mL^−1^, whereas the MIC value of 1,8-cineole was greater than 2 μL·mL^−1^. The results of this study also indicated that linalool was more active than 1,8-cineole on the tested bacteria. The bactericidal activities of 23 EO components were determined as BA_50_ values that represent a 50% decrease in the number of colony forming units (cfus) [3]. Although, results changed according to the tested organisms and components; BA_50_ values changed between 0.10–0.56 and 0.35–0.67 for α-terpineol and linalool, respectively and were higher than 0.67 for 1,8-cineole.

When α-pinene and 1,8-cineole were tested against four human pathogenic bacteria (*S. aureus*, *Enterococcus faecalis*, *E. coli* and *K. pneumonia*), the MIC values changed between 0.8 μL·mL^−1^ and 20 μL·mL^−1^ [31]. α-Pinene was found to be active against four bacteria, while 1,8-cineole showed no activity against *S. aureus* and *E. faecalis*, in contrast to our results. Components such as α-pinene is characterized by its bridged bicyclic structure. For α-pinene, MICs include 1.25 to 2.25 mg·mL^−1^ (0.12% to 0.22%) for *S. aureus*, *B. subtilis*, *P. aeruginosa* and *E. coli* [4]. In another study, α-pinene did not show any growth inhibitory activity against the tested bacteria, supporting our results [32].

In general, EO components can be subdivided into two distinct groups of chemical constituents; the hydrocarbons which are made up almost exclusively of terpenes (monoterpenes, sesquiterpenes, and diterpenes), and the oxygenated compounds (oxygenated terpenoids) which are mainly esters, aldehydes, ketones, alcohols, phenols, and oxides. Studies showed that oxygenated terpenoids such as alcoholic and phenolic terpenes have more antimicrobial activity than the other constituents. Several reports on the antimicrobial activity of monoterpenes have shown that the number of double bonds in a structure and the acyclic, monocyclic and/or bicyclic structure have no significant influence on their activity, although higher inhibitory activity is seen in aromatic compounds such as carvacrol, thymol and eugenol. However, oxygenated terpenoids show characteristic and distinct activity patterns towards microorganisms; terpenoids that contain alcohols possess higher activity than the corresponding carbonyl compounds [33,34,35,36].

As a result of their lipophilic character, monoterpenes will preferentially partition from an aqueous phase into membrane structures [34,35]. This results in membrane expansion, increased membrane fluidity and permeability, disturbance of membrane-embedded proteins, inhibition of respiration, and alteration of ion transport processes. Helander *et al*. [36] have described the effects of selected EO components on outer membrane permeability in Gram-negative bacteria, evidencing that monoterpene uptake is also determined by the permeability of the outer envelope of the target microorganism. However, the specific mechanisms involved in the antimicrobial action of monoterpenes remain poorly characterized.

There is some research regarding the antimicrobial activities of EO constituents. However, there has been no study concerning the synergistic effects of the components selected in this study. Combinations of terpenes such as thymol, carvacrol, eugenol, menthol, *etc*. [37,38,39], combinations of EOs [40,41,42], combinations of EOs/phenolics and nisin/bacteriocin [43,44] and also combination of EO components and food processing technique [45] were mostly studied. 

### 2.3. Synergism Testing of Constituents of Essential Oils

Three pathogenic bacteria showed more sensitivity to EO constituents. Therefore, the interactions between the constituents were examined against these bacteria. Checkerboard assays of all three tested bacteria gave additive or synergistic profiles when components were combined at sub-inhibitory concentrations (Table 3).

Synergistic effects were observed at least one dose pair of combination against *S*. Typhimurium, *E. coli* O157:H7 and *S. aureus* (data not shown). Synergy was noted when the components of α-terpineol and linalool were combined at ^1^/_4_ MIC + ^1^/_4_ MIC; ^1^/_4_ MIC + ^1^/_8_ MIC; ^1^/_8_ MIC + ^1^/_4_ MIC and ^1^/_8_ MIC + ^1^/_8_ MIC, respectively.

**Table 3:** Effect of Treatments with Combined Components According to FIC Index.

Bacterium	Components		MIC (%)		Combined MIC (%)		FIC	Effect
	A	B	A	B	A	B		
***E. coli* O157:H7**	*α-terpineol*	*linalool*	0.6	0.6	0.3	0.3	1	*Additive*
	*α-terpineol*	*linalool*	0.6	0.6	0.15	0.15	0.5	*Synergy*
	*α-terpineol*	*eucalyptol*	0.6	0.7	0.3	0.35	1	*Additive*
	*linalool*	*eucalyptol*	0.6	0.7	0.3	0.35	1	*Additive*
***S*** **. Typhimurium**	*α-terpineol*	*linalool*	0.7	0.7	0.35	0.35	1	*Additive*
	*α-terpineol*	*linalool*	0.7	0.7	0.175	0.175	0.5	*Synergy*
	*α-terpineol*	*eucalyptol*	0.7	0.8	0.35	0.4	1	*Additive*
	*linalool*	*eucalyptol*	0.7	0.8	0.35	0.4	1	*Additive*
***S. aureus***	*α-terpineol*	*linalool*	0.7	1	0.35	0.5	1	*Additive*
	*α-terpineol*	*linalool*	0.7	1	0.175	0.25	0.5	*Synergy*
	*α-terpineol*	*eucalyptol*	0.7	2	0.35	1	1	*Additive*
	*linalool*	*eucalyptol*	1	2	0.35	1	1	*Additive*

**Figure 2:**
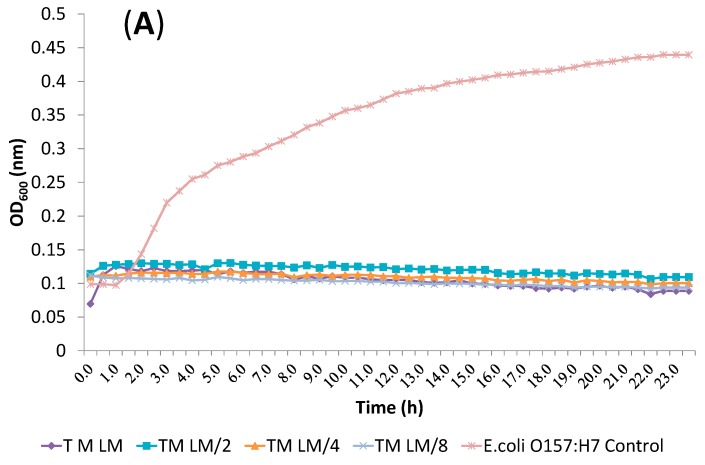

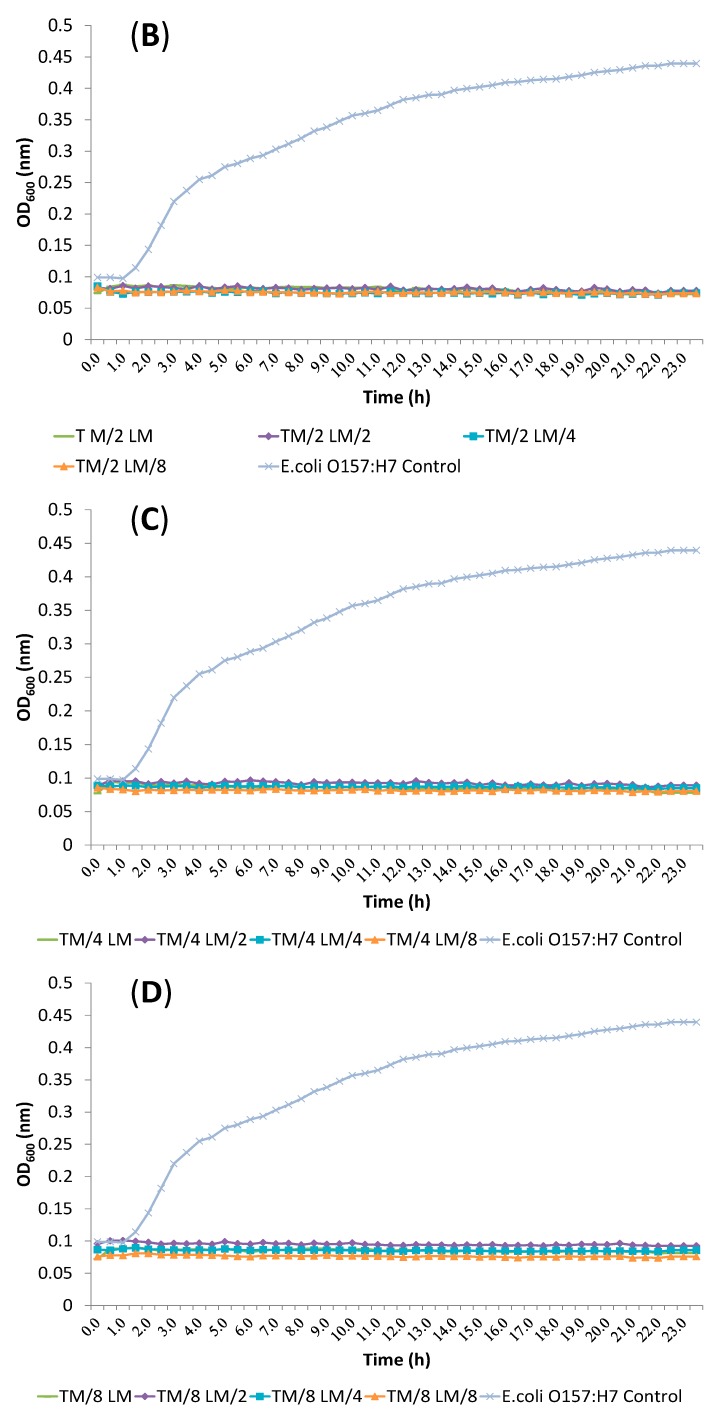
Inhibition of *E. coli* O157:H7 treated with α-terpineol/linalool (T/L) combinations at FIC index value. (**A**) TM *vs.* LM, L^M^/_2_, L^M^/_4_, L^M^/_8_; (**B**) T^M^/_2_ *vs.* LM, L^M^/_2_, L^M^/_4_, L^M^/_8_; (**C**) T^M^/_4_ *vs.* LM, L^M^/_2_, L^M^/_4_, L^M^/_8_; (**D**) T^M^/_8_ *vs.* LM, L^M^/_2_, L^M^/_4_, L^M^/_8_ (T: α-Terpineol, L: Linalool, M: MIC value, ^M^/_2_: MIC value/2, ^M^/_4_: MIC value/4, ^M^/_8_: MIC value/8).

**Figure 3:**
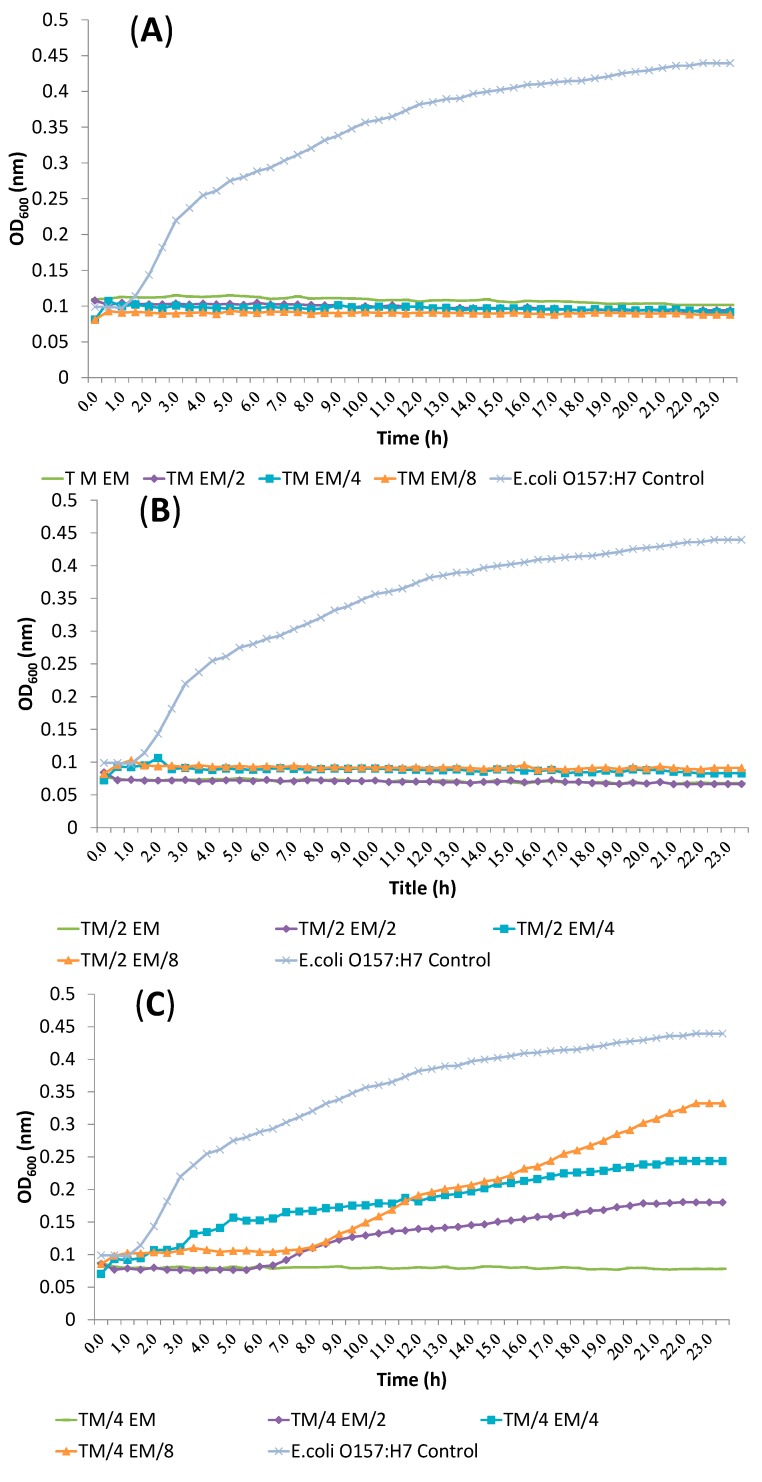

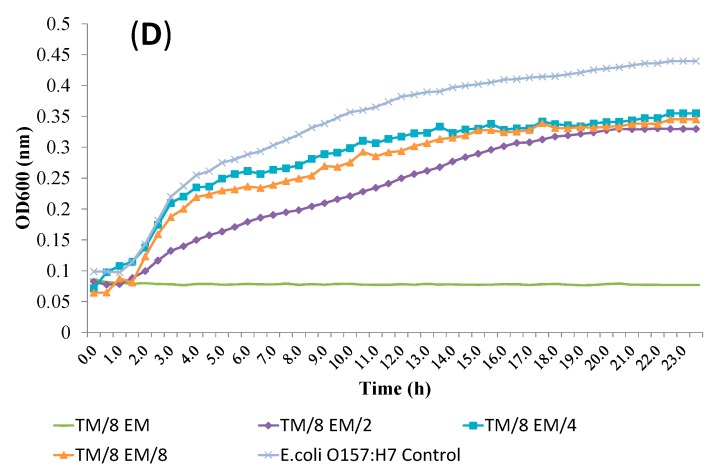
Inhibition of *E. coli* O157:H7 treated with α-terpineol/eucalyptol (T/E) combination at FIC index values. (**A**) TM *vs.* EM, E^M^/_2_, E^M^/_4_, E^M^/_8_; (**B**) T^M^/_2_ *vs.* EM, E^M^/_2_, E^M^/_4_, E^M^/_8_; (**C**) T^M^/_4_ *vs.* EM, E^M^/_2_, E^M^/_4_, E^M^/_8_; (**D**) T^M^/_8_ *vs.* EM, E^M^/_2_, E^M^/_4_, E^M^/_8_ (T: α-Terpineol, E: Eucalyptol, M: MIC value, ^M^/_2_: MIC value/2, ^M^/_4_: MIC value/4, ^M^/_8_: MIC value/8).

**Figure 4:**
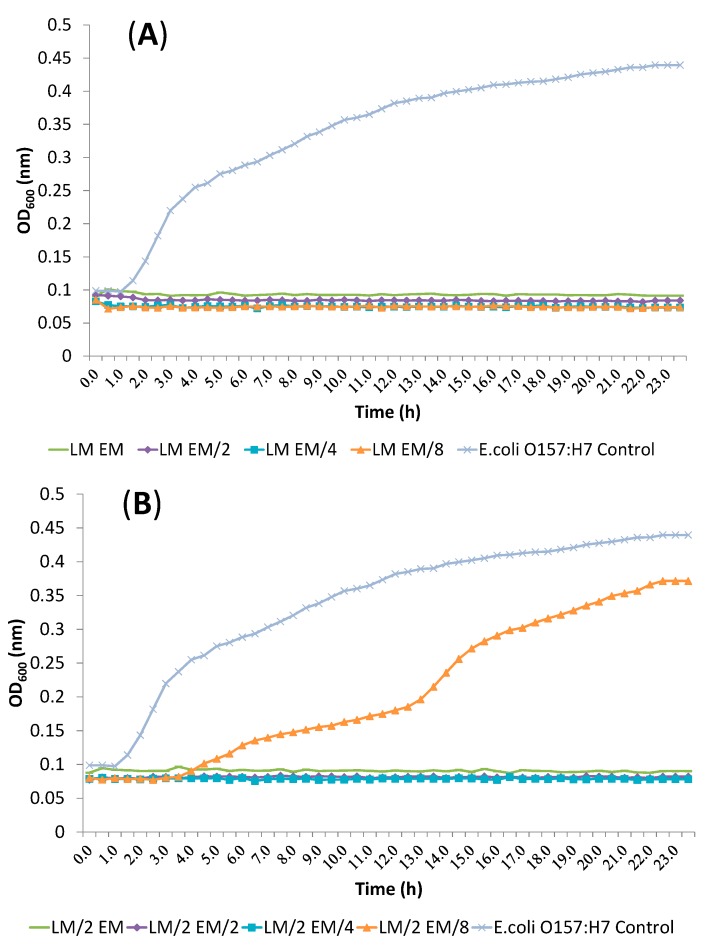

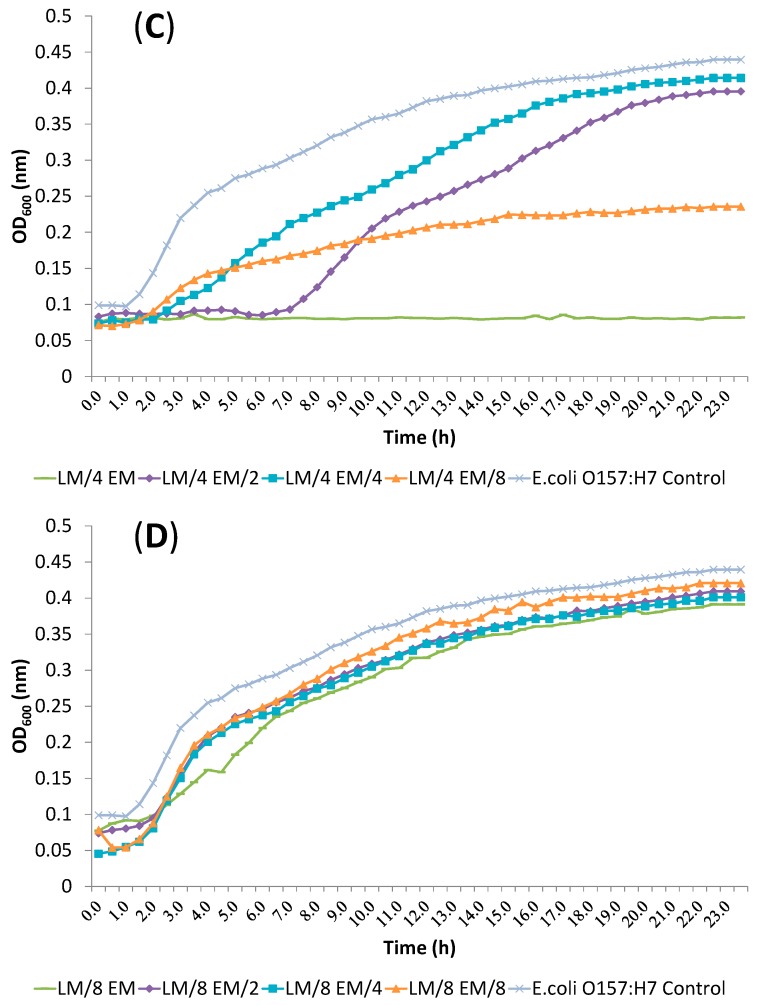
Inhibition of *E. coli* O157:H7 treated with linalool/eucalyptol (L/E) combination at FIC index values. (**A**) LM *vs.* EM, E^M^/_2_, E^M^/_4_, E^M^/_8_; (**B**) L^M^/_2_ *vs.* EM, E^M^/_2_, E^M^/_4_, E^M^/_8_; (**C**) L^M^/_4_ *vs.* EM, E^M^/_2_, E^M^/_4_, E^M^/_8_; (**D**) L^M^/_8_ *vs.* EM, E^M^/_2_, E^M^/_4_, E^M^/_8_ (L: Linalool, E: Eucalyptol, M: MIC value, ^M^/_2_: MIC value/2, ^M^/_4_: MIC value/4, ^M^/_8_: MIC value/8).

All other dose pair combinations resulted in additive effects. α-terpineol/eucalyptol and linalool/eucalyptol combinations showed additive effects against all tested bacteria. Inhibition of *E. coli* O157:H7 treated with α-terpineol/linalool (T/L), α-terpineol/eucalyptol (T/E) and linalool/eucalyptol (L/E) combination at FIC index values is shown in Figure 2, Figure 3 and Figure 4, respectvely.

Antagonistic effects were not found in these combinations. Most of the antimicrobial activity in EOs is found in the oxygenated terpenoids (e.g., alcohols and phenolic terpenes), while some hydrocarbons also exhibit antimicrobial effects [5,46]. Different terpenoid components of EOs can interact to either reduce or increase antimicrobial efficacy [47]. Interactions between these components may lead to antagonistic, additive or synergistic effects [5,46]. An additive effect is observed when the combined effect is equal to the sum of the individual effects. Antagonism is observed when the effect of one or both compounds is less when they are applied together than when individually applied. Synergism is observed when the effect of the combined substances is greater than the sum of the individual effects [5] while the absence of interaction is defined as indifference. Some studies have demonstrated that whole EOs usually have higher antibacterial activity than the mixtures of their major components, suggesting that the minor components are critical to the synergistic activity, though antagonistic and additive effects have also been observed [46]. Usually combinations, either single EOs or artificial mixtures of purified main components, affect multiple biochemical processes in the bacteria, producing a plethora of interactive antibacterial effects [46].

Interactions reported for terpene comminations of thymol/carvacrol; thymol/eugenol; carvacrol/linalool; eugenol/linalool, eugenol/menthol, 1,8-cineole/aromadendrene; limonene/1,8-cineole; α-pinene/limonene, α-pinene/linalool, linalool/terpinen-4-ol and cinnamaldehyde/carvacrol were additive, synergism, antagonism [12,46]; synergism [39]; additive [48]; synergism [46]; synergism, additive [46]; additive, synergism [39,46], respectively.

In basil, the strongest antimicrobial activity of sweet basil was attributed to the eugenol (19%) and linalool (54%) content and a synergistic effect was observed. The importance of the hydroxyl group (-OH) of phenols was demonstrated by the higher antimicrobial and antioxidant activities of eugenol in relation to methyleugenol (-O-Me) [49]. Lis-Balchin and Deans [50] showed that EOs containing large amounts of 1,8-cineole were better antilisterial agents than EOs devoid of it.

Interestingly, phenolic monoterpenes and phenylpropanoids (typically showing strong antimicrobial activities) in combination with other components were found to increase the bioactivities of these mixtures. Most of the studies have focused on the interaction of phenolic monoterpenes (thymol, carvacrol) and phenylpropanoids (eugenol) with other groups of components, particularly with other phenols, phenylpropanoids and monoterpenes alcohols, while monoterpenes and sesquiterpenes hydrocarbons were used to a lesser extent. The combination of phenolics with monoterpenes alcohols produced synergistic effects on several microorganisms, in particular, the combination of phenolics (thymol with carvacrol, and both components with eugenol) were synergistically active against *E. coli* strains, though other reports have observed additive [12] and antagonism effects [38].

### 2.4. Release of Cellular Material after Treatment with Essential Oil Components

**Figure 5:**
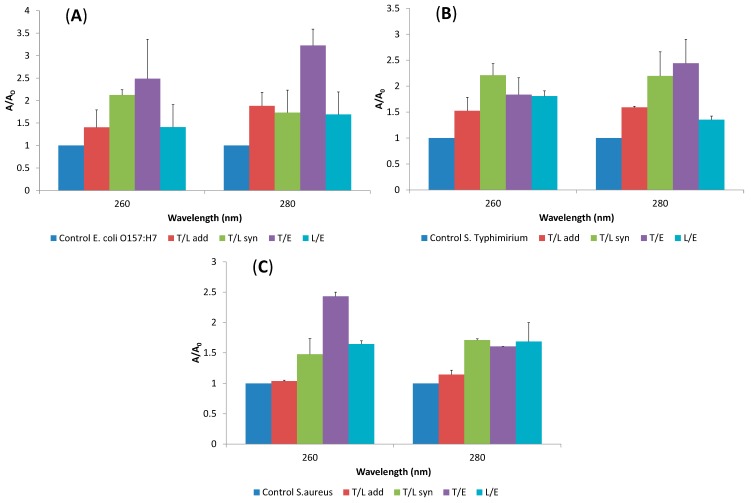
Release of cellular material at 260-nm and 280-nm for *E. coli* O157:H7 (**A**); *S*. Typhimurium (**B**); and *S. aureus* (**C**) cells treated with combinations at FIC values. (T: α-Terpineol, L: Linalool, E: Eucalyptol, add: additive; syn: synergy).

Another strategy for determining the mode of action of EO components against tested bacteria was performed on the basis of release cell constituents determined by the measurement of the absorbance at 260 nm and 280 nm of the supernatant of three tested treated strains. Figure 5 shows the cell constituents release test results when *S. aureus*, *E. coli* O157:H7 and *S.* Typhimurium were treated with EO components at FIC values for 2 h, respectively.

The results indicated that after exposure to the corresponding EO components, the cell constituents’ release increased visibly compared to the control group. When *E. coli* O157:H7 cells were treated with α-terpineol and eucalyptol combination at the FIC value of an additive effect, the highest release of 260 nm and 280 nm absorbing materials was observed (Figure 5A). The treatment with α-terpineol and linalool at FIC value of additive effect was resulted in higher 280 nm absorbing materials than 260 nm, whereas, treatment with a synergistic effect FIC value caused lower absorbing materials at 280 nm than 260 nm. Linalool and eucalyptol combination led to nearly the same release for both 260 nm and 280 nm absorbing materials.

The release of cellular material from *S.* Typhimurium were nearly the same for both 260 nm and 280 nm absorbing materials for the treatments with α-terpineol and linalool at FIC value of additive and synergistic effects (Figure 5B). α-terpineol and eucalyptol combination caused a higher release of 280 nm absorbing material than 260 nm. *S. aureus* cells showed higher release for 260 nm absorbing material than 280 nm for the treatment of α-terpineol and eucalyptol combination (Figure 5C). Whereas, at 280 nm absorbing material for α-terpineol and linalool combination was seemed to be higher than that of absorbing material at 260 nm. The other combinations led to the nearly the same amount of release for both 260 nm and 280 nm absorbing material. It was also observed that the 280 nm absorbing material releases were nearly the same for all combination pairs. The results of release of cellular materials test showed that α-terpineol/linalool, α-terpineol/eucalyptol and linalool/eucalyptol combination treatments have a strong effect on the release of cell constituents both from Gram negative and positive bacteria.

As seen in Figure 5 and Table 3 α-terpineol/eucalyptol combination which was resulted in additive interaction demonstrated a higher effect on the release of cellular materials (at both 260 and 280 nm) of *E. coli* O157:H7 followed by *S.* Typhimurium and *S. aureus* cells. However, α-terpineol/linalool combination causing synergistic interaction exerted approximately the similar cell constituent release effect on these bacteria.

It can be concluded that eucalyptol has more effect individually than the other terpenes on the release of cellular materials from bacterial cells. In general *E. coli* O157:H7 and *S.* Typhimurium treated with all the terpene combinations displayed higher leakage of the cell content than the *S. aureus* cells which could be attributed to the differences in the outer membrane and cell wall structures.

Similarly Lv *et al.* [7] determined higher cell constituents release (260 nm) from *E. coli* than *S. aureus* cells treated with EO combinations. However, in the same study maximum cell constituents release was observed for Gram positive *Bacillus subtilis* [7].

In the related literature, loss of cell constituents including proteins, K^+^, nucleic acids, ATP and enzymes (transaminase) of both Gram positive and negative bacteria have been observed after treatment with EOs or their components [7,12,44,51]. However, these findings have not been able to precisely explain the effects of EOs and their components on the specific intracellular constituents such as proteins.

### 2.5. SEM Microscopy

Pathogenic bacteria (*S. aureus*, *S.* Typhimurium, *E. coli* O157:H7) were treated for 2 h with each EO terpene components (eucalyptol, α-terpineol and linalool) using relevant MIC values. They were then observed by SEM to investigate the resulting morphological changes in the appearance of the cells.

**Figure 6:**
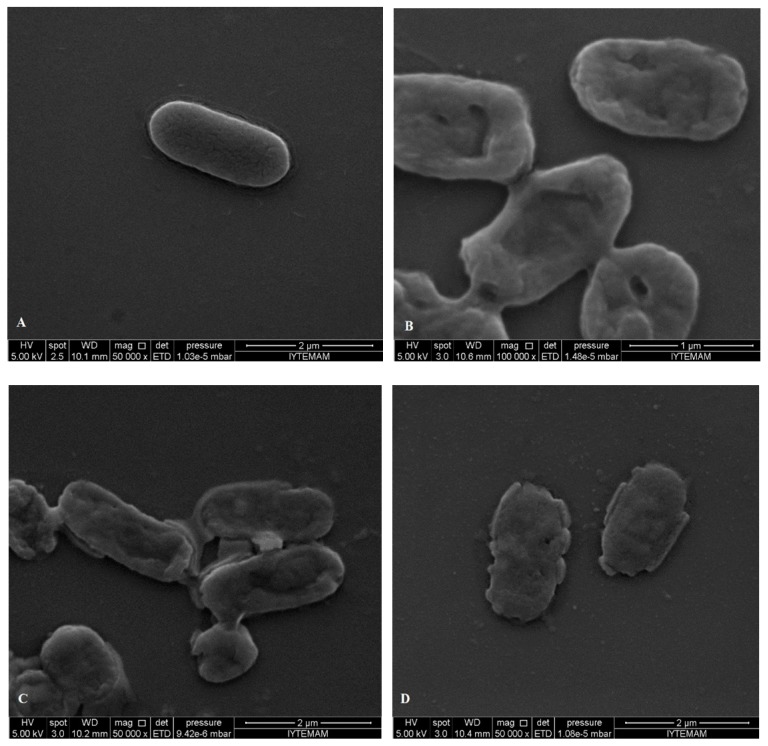
SEM micrographs of untreated bacterial cells of (**A**) *E. coli* O157:H7; treated bacterial cells of *E. coli* O157:H7 with (**B**) eucalyptol; (**C**) linalool; (**D**) α-terpineol.

**Figure 7:**
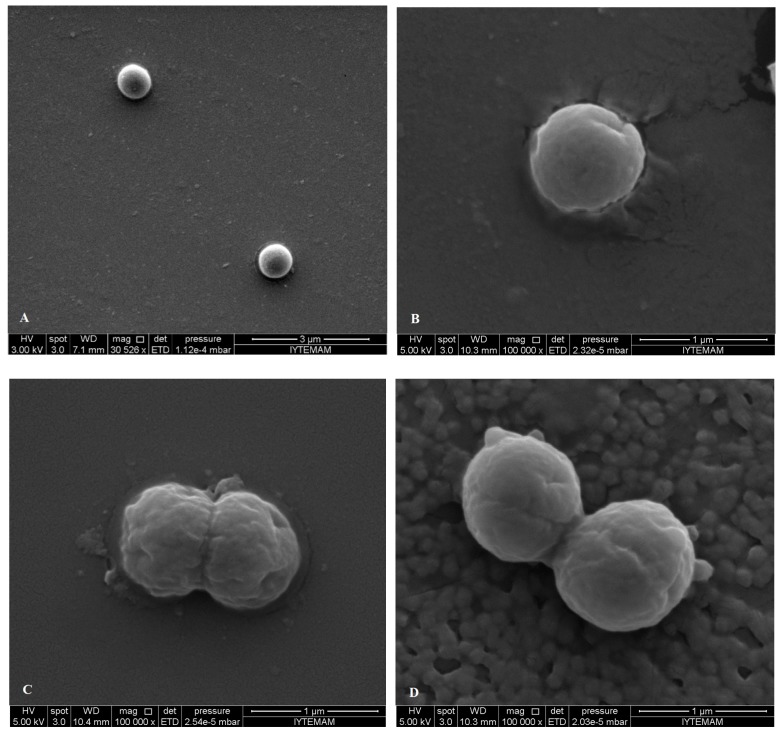
SEM micrographs of untreated bacterial cells of (**A**) *S. aureus*; treated bacterial cells of *S. aureus* with (**B**) eucalyptol; (**C**) linalool; (**D**) α-terpineol.

SEM images showed differences in cell structures between terpene-treated bacteria and the non-treated control bacteria. Non-treated cells were intact (regular rod or coccus shaped) and showed a smooth surfaces as can be seen in Figure 6A and Figure 7A, while bacterial cells treated with the individual terpenes underwent considerable structural changes as can be obviously discriminated in Figure 6B–D and Figure 7B–D. SEM observations confirmed the damage to the structural integrity of the cells and considerable morphological alteration to all tested Gram-positive and Gram-negative bacteria. In Figure 6 it is obvious that treatment with the terpenes caused pores on the outer membrane of *E. coli* O157:H7 cells which enabled the cell constituents to pass easily through these and also caused collapsing of the cells. The outer membrane or the cell wall of the bacteria is most likely to be the cellular target for terpenes due to the formation of perforations. Moreover, differences in cell wall composition (more or less lipids) between different bacterial species partially accounts for their differential susceptibility to terpenes. Eucalyptol, α-terpineol and linalool terpene components caused permeability alteration of the outer membrane, alteration of cell membrane function and leakage of intracellular materials. This was also supported by the our results from the cell constituent release tests.

There are many possible explanations for the observations. Some authors have suggested that the damage to the cell wall and cytoplasmic membrane was the loss of structural integrity [52,53,54]. The literature suggests that the active components of the plant extracts might bind to the cell surface and then penetrate to the target sites, possibly the phospholipid bilayer of the cytoplasmic membrane and membrane-bound enzymes [55]. In addition to interacting with membrane phospholipids, interaction with membrane proteins and intracellular targets is also suggested [56]. The effects might include the inhibition of proton motive force, inhibition of the respiratory chain and electron transfer, and inhibition of substrate oxidation. Uncoupling of oxidative phosphorylation, inhibition of active transport, loss of pool metabolites, and disruption of synthesis of DNA, RNA, protein, lipid, and polysaccharides might follow [8,10]. These images confirm the loss of shape and integrity which was followed by the cell death. Cell death may have been the result of the extensive loss of cell contents, the exit of critical molecules and ions, or the initiation of autolytic processes [57].

**Figure 8:**
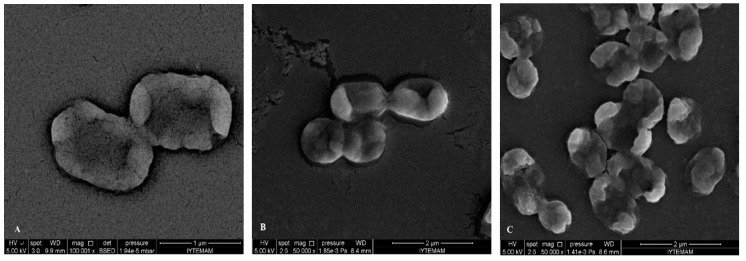
SEM micrographs of treated *E. coli* O157:H7 cells with combination of the terpenes at FIC values (with their effect): (**A**) α-terpineol/linalool; (**B**) α-terpineol/eucalyptol; (**C**) linalool/eucalyptol (*additive*).

**Figure 9:**
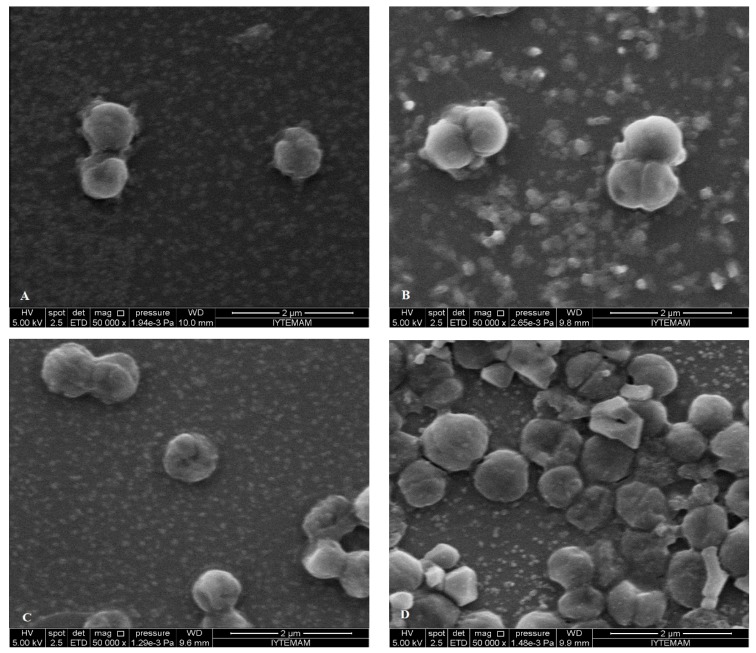
SEM micrographs of treated *S. aureus* cells with combination of the terpenes at FIC values (with their effect): (**A**) α-terpineol/linalool (*additive*); (**B**) α-terpineol/linalool (*synergistic*); (**C**) linalool/eucalyptol (*additive*); (**D**) α-terpineol /eucalyptol (*additive*).

After treatment and observation of bacterial cells with each component, FIC values determined by checkerboard assay were used to treat bacteria and observation with SEM. When SEM images of FIC treated bacteria and component treated ones, it was observed that FIC treated bacteria were highly damaged. Cells treated with components at FIC concentrations revealed severe damaging effect on the cell morphology of the tested pathogens, showing large surface collapse and abnormal cell breaking, as well as complete lysis or dead cell formation (Figure 8 and Figure 9). Combined terpene treatments altered outer membrane and the structures of the cells and made them more permeable. This disrupted osmotic balance causing more leakage of cellular molecules. Bacterial cell wall or peptidoglycan is a cross-linked mesh that gives a cell its shape, strength and osmotic stability. Therefore, loose of regular shape, structural integrity and osmotic stability after combined terpene treatment confirms the outer membrane or the cell wall of the bacteria most likely be cellular target for terpenes.

Combined terpene treatments altered the outer membrane and the structures of the cells and made them more permeable. This disrupted osmotic balance causing more leakage of cellular molecules. Bacterial cell wall or peptidoglycan is a cross-linked mesh that gives a cell its shape, strength and osmotic stability. Therefore, loss of regular shape, structural integrity and osmotic stability after combined terpene treatment confirm the outer membrane or the cell wall of the bacteria to be the most likely cellular target for terpenes.

The combined synergistic interaction of the monocyclic α-terpineol and acyclic linalool, which chemically are monoterpene alcohols and both have hydroxyl groups (-OH) may also be responsible for their interaction with intracellular components. Combined treatments of eucalyptol (1,8-cineole) which is chemically a a monoterpene ether with monoterpene alcohols (α-terpineol and linalool) resulted in additive interaction on the both Gram positive and negative cells. This additive effect was seen as structural deformation, breakage of cell wall and outer membrane, release of intracellular material from the damaged cells of *E. coli* O157:H7 and were observed as irregular and shriveled cell structures, leakage of the contents of the cells and lysis of *S. aureus* in the Figure 8B,C and Figure 9C,D, respectively*.* Our results confirm the importance of the -OH in the antimicrobial mode of action of terpene alcohols, as reported by others [49,50].

In the literature there are few reports on the antimicrobial mechanisms of action of combinations of EOs or their purified components on microorganisms [5,33,46]. Some publications deal with the mode of action of the EO components in combination with other natural preservatives or antibiotics [46,50]. Although there are limited numbers of papers dealing with the antimicrobial mechanism of action of combinations of the EOs or their components, antimicrobial interaction that produces synergism is one of the mechanisms studied. Possible mechanisms include the sequential inhibition of a common biochemical pathway, inhibition of protective enzymes and use of cell wall active agents to enhance the uptake of other antimicrobials [46]. Synergism between carvacrol and some monoterpene hydrocarbons (e.g., α-pinene, camphene, myrcene, α-terpinene and *p*-cymene) that typically showed low antimicrobial properties has been observed [40,58]. The capacity of hydrocarbons to interact with cell membrane facilitates the penetration of carvacrol into the cell [40,51,58].

There are many reports of the antibacterial activities of plant-derived compounds, such as EOs. In general, Gram-positive bacteria are more sensitive to terpenes than Gram-negative ones and this was also confirmed in our study. Moreover, the Gram-negative species *S. putrefaciens* was found to be the most resistant bacterium to the terpenes. However, to our knowledge, there is no report with which to compare this finding. Differences in the permeability, composition, and charge of the outer structures of the microorganisms determine the sensitivity to terpenes. Monoterpenes preferentially influence membrane structures which increases membrane fluidity and permeability, changing the topology of membrane proteins and inducing disturbances in the respiration chain [33]. The external structure of the outer membrane of *E. coli* and *Salmonella* is almost entirely composed of lipopolysaccharides (LPS) and proteins. These bacteria have a hydrophilic surface due to the O-side chains of the LPS, making it difficult for hydrophobic molecules, like lipids, to enter the bilayer.

In addition, molecules may have difficulty in penetrating outer membranes because of the low fluidity of the hydrocarbon chains in the LPS structure and the strong interactions between the LPS molecules. However, it has been shown that highly lipophilic compounds penetrate easily through the outer membrane of the bacteria.

Although the antibacterial mode of action of terpene is still entirely unknown, our results confirmed that it is most probable the lipophilicity and/or hydrophobicity of the terpene and presence of hydroxyl group (-OH) in the components have great effects on the mechanism of their antibacterial action.

To clarify the mode of action of such activities, each constituent should be assayed separately. Our results reveal that terpenes, which are a major category of plant-derived compounds, might interact with each other and with bacterial cells to increase or decrease each other’s antibacterial activity. Therefore, interactions between terpene components lead to additive or synergistic effects. Thus, it is important to investigate not only single constituents but also combinations in studies of the antibacterial activities of plant-derived compounds. Further studies are needed to characterize the mechanisms of changes in antibacterial activities in systems that include mixtures of terpenes.

## 3. Experimental Section

### 3.1. Essential Oil Components

All essential oil (EO) components, namely eucalyptol, linalool, α-terpineol and α-pinene were obtained from Sigma-Aldrich (St. Louis, MO, USA). All the chemicals used in the study were of analytical grade and they were purchased from Sigma Chemical Co. (St. Louis, MO, USA).

### 3.2. Bacterial Strains

The antibacterial activity of EO components (eucalyptol, linalool, α-terpineol and α-pinene) was tested against bacterial strains, of which eight were reference strains: four Gram-negative strains (*Escherichia coli* O157:H7 ATCC700728, *Salmonella* Typhimurium CCM5445, *Shewanella putrefaciens* NRRLB-951 and ground beef isolate *Serratia liquefaciens* NRRL B-41553), three Gram-positive strains (clinical isolate *Staphylococcus aureus* RSSK01009, turkey/ham isolate *Listeria innocua* NRRLB-33314, minced-beef isolate *Carnobacterium divergens* NRRLB-14830). *S. putrefaciens*, *C. divergens* and *S. liquefaciens* strains were obtained from USDA, ARS Culture Collection. *S. aureus* and *L. innocua* strains were kindly provided by Dr. F. Korel (Department of Food Engineering, İzmir Institute of Technology) and Dr. N. Demirel Zorba (Department of Food Engineering, Çanakkale Onsekiz Mart University), respectively.

*E. coli* O157:H7, *S.* Typhimurium, *S. aureus* and *L. innocua* strains were grown in Nutrient Broth (NB, Difco, Becton, Dickinson and Co., Franklin Lakes, NJ, US). *S. putrefaciens* and *S. liquefaciens* were firstly propagated in Tryptic Soy Broth (TSB, Difco, BD), whereas *C. divergens* was grown in YG broth (25 g nutrient broth no:2, 3 g yeast extract, 5 g glucose, 1 L of water; pH 6.8). After first propagation all bacteria were transferred to NB and the growth was observed. NB medium was used for all the experiments. All bacterial strains were preserved in NB containing 20% (v/v) glycerol at −80 °C.

### 3.3. Preparation of Bacterial Suspensions

Cultures were grown in appropriate media and at optimum incubation conditions. Bacterial suspensions were adjusted equivalent to 0.5 McFarland standard (~10^7^–10^8^ cfu·mL^−1^) (Densitometer, DEN-1, HVD Life Sciences, Vienna, Austria) and one more tenfold dilution was made in NB medium.

### 3.4. Determination of Antioxidant Activity by the FRAP Method

Ferric reducing antioxidant power (FRAP) assays were performed according to Thaipong *et al.* [59]. The fresh working FRAP solution was prepared by mixing acetate buffer (10 mL, 300 mM, pH 3.6), 2,4,6-tripyridyl-s-triazine (TPTZ, 1 mL, 10 mM) and FeCl_3_·6H_2_O solution (1 mL) and then warming at 37 °C before use. Diluted EO components (150 µL) were allowed to react in the FRAP solution in a 3 mL total volume for 30 min in the dark condition. Readings were then taken at 593 nm and results were expressed as millimol Trolox equivalent·mL^−1^.

### 3.5. Determination of Antioxidant Activity by the DPPH Method

DPPH radical was determined by the method described in [31] with some modifications. Briefly each sample (20 µL) in triplicate and six different concentrations and DPPH solution (180 µL, 160 mM) in ethanol, were added to a well in a 96-well flat-bottom microtitration plate. A DPPH solution was used as blank sample. Plates were incubated for 24 h and absorbance was measured at 515 nm with 10 min time intervals. The antioxidant activity of the tested samples, expressed as percentage inhibition of DPPH, was calculated according to the formula:

IC (%) = [(Ab − As)/Ab] × 100

where Ab = absorbance of blank sample and As = absorbance of a tested sample at the end of the reaction [31,60]. Percent inhibition after where the reaction gone to completion (“plateau”) was plotted against concentration and a linear regression was applied to obtain the IC_50_ value [61].

### 3.6. Minimal Inhibitory Concentration (MIC) by Broth Micro- and Macro-Dilution Method

For broth microdilution, bacterial suspension (20 μL) was added to the wells of a sterile 96-well microtitre plate containing EOs or components (180 μL). Control wells were prepared with culture medium inoculated with bacterial suspension and compounds without inoculation. Plates were incubated for 24 h and the turbidity was determined by a microplate reader (Varioskan^®^ Flash, Thermo Fisher Scientific Inc., Waltham, MA, USA) at 600 nm with 30 min intervals [62]. The MICs of EOs or components were recorded as the lowest concentration where no visible growth was observed in the wells after incubation for 24 h. For broth macrodilution bacterial suspensions (100 μL) were inoculated into growth media (900 μL) already containing the desired concentration of EO component and incubated by shaking for 24 h at appropriate incubation temperatures. After 24 h 100 μL sample was directly spread on agar plates and growth of colonies was checked after incubation for 24–48 h.

### 3.7. Synergism Testing of Terpenes by Checkerboard Method

The antimicrobial activity of eucalyptol, linalool, α-terpineol and α-pinene was analyzed alone or in combination to evaluate the potential interaction among them. The checkerboard assay was performed to determine potential synergistic, additive or even antagonistic effects of combination of individual compounds by defining Fractional Inhibitory Concentration [63] index using 96-well microtitre plates [37,48]. The combinations were designed by using the concentration of constituents ranging from MIC to 1/8 MIC. The final volume of each well was 100 μL including 50 μL of each constituent dilution. Subsequently, bacterial suspensions (100 μL) were added to the wells. The plates were then incubated at 37 °C for 24 h and the turbidity was determined by a microplate reader (Varioskan^®^ Flash, Thermo Fisher Scientific Inc.) at 600 nm with 30 min intervals. The FIC indices were calculated as FICA + FICB where each of them was the minimum concentrations that inhibited the bacterial growth. FIC index was calculated as follows;

FICA = MIC of component A in combination/MIC of component A alone

FICB = MIC of component B in combination/MIC of component B alone

FIC = FICA + FICB

The combination of two compounds was considered to be synergistic when the FIC index was ≤0.5, additive when it was >0.5 to 4 and antagonistic when it was >4 [40,64,65].

### 3.8. Release of Cellular Material

Overnight broth cultures were adjusted to McFarland 2 standard. Cells were collected by centrifugation at 5000 rpm for 5 min. The pellet was washed twice and resuspended in PBS (pH 7.2) containing FIC values of essential oil components. Samples were incubated at appropriate temperature for each bacterium under agitation for 1 or 2 h. After treatment cell suspensions were centrifuged at 10,000 rpm for 10 min. Supernatant was used to measure UV absorptions at both 260 and 280 nm to determine the concentration of the cell constituents released [7,66]. Results were expressed as the ratio of OD_260_ and OD_280_ of incubated samples to initial measurements of OD_260_ and OD_280_, respectively.

### 3.9. SEM Microscopy

Overnight cultures were adjusted to McFarland 1 standard and then treated with EO constituents at the determined FIC values. After appropriate incubation period for each bacterium, cells were harvested by centrifugation, and washed 2 or 3 times and resuspended in sterile distilled water or PBS. 20 μL of suspension was spread onto a microscope slide and air dried. Another portion of cultures were used as untreated control. Then samples were coated with gold under vacuum followed by microscopic examinations using SEM (XL 30 SFEG, Philips Electronic Optics, Eindhoven, The Netherlands) [1,7].

### 3.10. Statistical Analysis

All the analysis were carried out in triplicate and the experimental results obtained were expressed as means ± SD. Means with a significant difference (*p* < 0.05) were compared using Tukey test (Minitab 16, Minitab Inc., State College, PA, USA).

## 4. Conclusions

The antimicrobial and antioxidant activities of the terpene EOs are highly affected by their chemical nature and interactions. This study showed that terpene EO components are effective on the biological activities by producing synergistic and additive effects on the common prominent pathogenic and spoilage food-related bacteria. In conclusion the characterization of the mechanisms of changes in antibacterial activities in systems that include mixtures of terpenes is needed to choose and to determine their concentrations for the best health purposes and food safety applications.
